# Painless Scrotal Ulcers Become Something Unexpected: A Rare Case of Scrotal Calciphylaxis

**DOI:** 10.7759/cureus.28958

**Published:** 2022-09-08

**Authors:** Riddhima Issar, Jinisha Patwa, Yvette Wang

**Affiliations:** 1 Internal Medicine, Rowan University School of Osteopathic Medicine, Stratford, USA

**Keywords:** painless cutaneous ulcers, dialysis, calciphylaxis, end stage renal disease, atypical presentation

## Abstract

Calciphylaxis is an uncommon vascular disorder that presents with painful skin necrosis due to calcium accumulation in the skin and adipose tissue. It often presents in patients with multiple comorbidities like end-stage renal disease (ESRD) and in patients who are on long-term dialysis. This case highlights the atypical presentation of painless ulceration as seen in our patient. A 68-year-old Caucasian male with a past medical history of ESRD on hemodialysis (HD), diabetes mellitus II, and peripheral vascular disease presented to the hospital with altered mental status and severe left foot necrosis. During the hospital course, the patient developed a painless scrotal wound and eschar with sloughing. The ulcer was non-tender to palpation. Ultrasound identified calcifications in the scrotal tissue and CT of the pelvis without contrast showed extensive calcification of the arterial system. A clinical diagnosis of calciphylaxis was made. Treatment was initiated with sodium thiosulfate. The patient stabilized over the next few days; however, a few days later, the patient was readmitted and unfortunately passed away due to cardiac arrest. This case delineates the atypical presentation of calciphylaxis. Although painful skin necrosis is a well-reported and classic presentation of this disease, the lack of pain perception despite such a severe condition in our patient is of particular interest. This case calls for a closer look into the diagnosis of calciphylaxis, especially in the presence of nontender skin ulcers. This diagnosis should be part of the differential in a patient with ESRD on HD even when the presentation is atypical as early diagnosis and intervention can prevent fatal outcomes.

## Introduction

Calciphylaxis is a rare and challenging vascular disorder characterized by painful cutaneous ulceration and necrosis. The pathophysiology of this disease is not well defined but several theories illustrate that skin ulcerations and necrosis are a result of reduced blood flow in the arterial system due to the presence of calcifications, sclerosing, and fibrosis [1]. Furthermore, an imbalance of calcium and phosphate metabolism has also been noted to play a role [1]. This occurs with underlying comorbidities like chronic kidney disease and its complications. This rare disease is fatal and carries a high risk of mortality. Several studies have shown that in patients with this disease, there is an estimated six-month survival of 50% [2].

The disease is challenging to recognize and especially challenging to treat. Recent trends in the United States revealed an increase in the prevalence of calciphylaxis in patients on hemodialysis [3]. The disease is often seen among patients with several comorbidities like end-stage renal disease (ESRD) [4], type 2 diabetes mellitus (T2DM) [5], and hyperparathyroidism (HD) [6]. Typically, the disease presents itself with painful cutaneous red indurations, nodules that progress to ulcers, skin necrosis, and eschars that are tender to palpation [6]. This increases the risk of developing gangrene and sepsis. Most commonly, cutaneous manifestations of calciphylaxis are seen on the buttocks, thighs, extremities, and in regions of high-density adipose tissue. 

Very few cases have presented unconventional locations of these calcified eschars and ulcers such as the penis and scrotum [7-11]. Several atypical presentations of calciphylaxis are reported in the literature; however, all delineate a similar clinical presentation of painful ulcerations and eschars [12]. Although the diagnosis of calciphylaxis is challenging and poses many concerns, it is essential to note that currently, a skin biopsy is the gold standard to diagnose this disease [6]. Various other imaging modalities and the comorbidity profile of a patient can help in the diagnosis of this disease. The current treatment approach for cutaneous manifestations includes wound care, pain management, and other supportive care [13]. Several case studies and series have suggested the use of sodium thiosulfate in patients with calciphylaxis [13]. Despite supportive measurements and pharmacotherapy, calciphylaxis has a severe disease progression with a one-year mortality rate between 45-80% [14]. Therefore, this disease carries a high mortality burden, which further increases the need to recognize atypical presentations of this disease. 

This article was previously presented as a poster in abstract form at the 2022 Society of General Internal Medicine Conference on April 6, 2022.

## Case presentation

**Figure 1:**
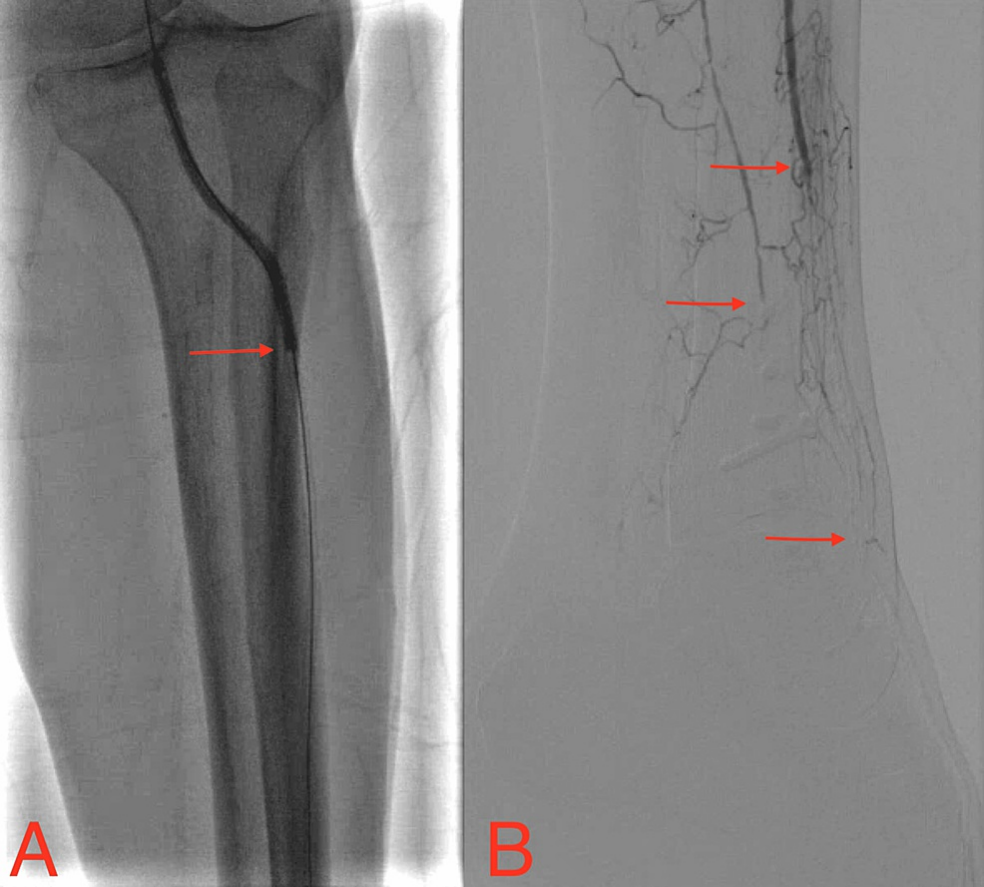
Left lower extremity arteriogram: (A) anterior view; (B) lateral view Arrows depict occluded and stenotic vessels.

We report an atypical case of painless scrotal calciphylaxis masked by neuropathy in an ESRD patient on HD. A 68-year-old Caucasian male with a past medical history of ESRD on HD, T2DM, peripheral vascular disease (PVD), and neuropathy presented to the hospital with altered mental status (AMS) and severe left foot necrosis. He was evaluated by vascular surgery and podiatry. Initial presentation of the left foot necrosis showed nonpalpable pulses on physical exam and Doppler ultrasonography. Furthermore, a left lower extremity arteriogram confirmed non-reconstructible PVD due to >50% stenosis in the left popliteal, and left anterior tibial artery; a fully occluded left posterior tibial, peroneal, plantar, and dorsalis pedis artery was seen (Figure 1). Therefore, below the knee amputation of the left leg was performed with an eventual unremarkable recovery in the hospital.

**Figure 2:**
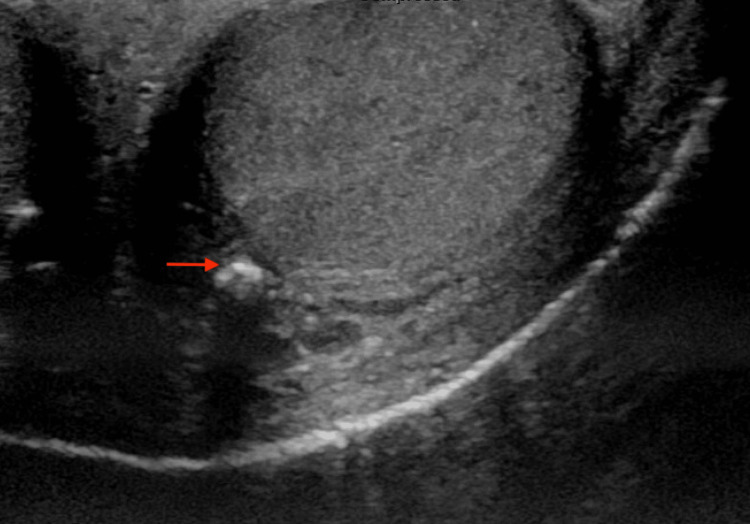
Scrotal ultrasound Arrow depicts calcifications identified on ultrasound

**Figure 3:**
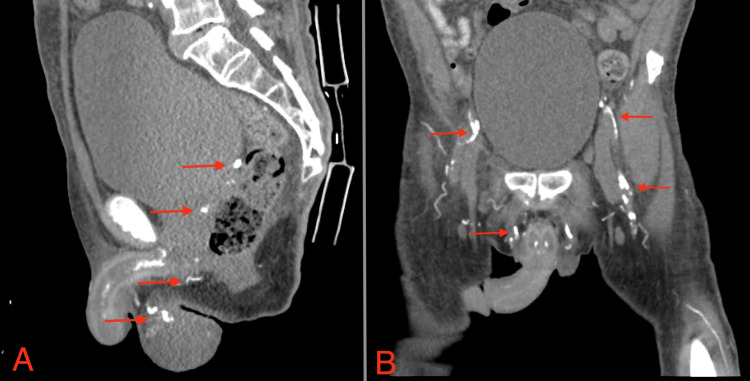
CT of the pelvis without contrast: (A) lateral view; (B) anterior view Arrows depict calcified content

A few days after the initial presentation, a painless 5 cm mid-scrotal wound and eschar with sloughing was noted on physical exam. No crepitus or fluctuance was noted. The ulcer was non-tender to palpation. A neurological exam showed no sensory deficits to light touch in the lower extremity bilaterally. Patient’s laboratory results showed calcium levels of 8.7mg/dL (normal: 8.5 to 10.2 mg/dL), an elevated phosphate of 7.3 mg/dL (normal: 2.5 to 4.5 mg/dL), and an elevated parathyroid hormone of 148 pg/mL (normal: 14 to 65 pg/mL). On the same day, ultrasound identified calcifications in the scrotal tissue (Figure 2), and a computed tomography (CT) scan of the pelvis without contrast showed extensive calcification of the arterial system (Figure 3). However, the scrotal biopsy indicated severe skin and soft tissue necrosis with acute and chronic inflammation; no calcium deposits were seen. A differential diagnosis of calciphylaxis versus Fournier's gangrene was considered. Fournier’s gangrene was ruled out by urology and nephrology due to the absence of systemic signs like fever and hemodynamic instability, absence of cutaneous signs like crepitus, nonspecific imaging findings of the deep tissue, and the absence of gas in radiographic images. Due to this low likelihood, surgical exploration for possible Fournier's gangrene was deferred. A clinical diagnosis of calciphylaxis was made given that the eschar raised suspicion for calciphylaxis along with the concerning ultrasound and CT findings, in association with the patient’s comorbidities of secondary hyperparathyroidism, ESRD, and his nonadherence to HD. Treatment was initiated with sodium thiosulfate 12.5 g intravenously with a maintenance dose titrated to 25 g. The patient stabilized over the next few days and made a remarkable recovery leading to an eventual discharge to a post-acute care facility. Five days later, the patient was readmitted for evaluation of AMS and unfortunately passed away due to cardiac arrest. 

## Discussion

Calciphylaxis, also known as calcific uremic arteriolopathy, is a rare diagnosis, seen most often in patients with chronic kidney disease and renal failure. It is especially prevalent in patients who are on HD. This disease presents with painful skin plaques or nodules, which eventually progress to painful necrosis, ulcers, and eschars. This case highlights the severity of a calciphylaxis diagnosis and the atypical presentation of painless ulcers along with the increased mortality rate that is associated with severe PVD. Although skin necrosis is typical of this disease, the lack of pain perception despite such a severe condition in our patient is noteworthy. As discussed earlier, a handful of cases have reported penile calciphylaxis [7-11]. Although all of these cases had similar risk factors (ESRD on HD, hyperparathyroidism, PVD) as our patient, these cases all had an initial presentation of painful and necrotic ulcers and eschars in stark contrast to our patient’s initial presentation. Furthermore, while the current literature provides data on several cases of penile calciphylaxis, scrotal calciphylaxis is seen less frequently and has little to no data on management and treatment when the presentation is painless. 

It is unclear why the patient did not experience any pain with his cutaneous lesions. Several theories can be made as to why the patient did not perceive pain upon palpation during the physical exam. One theory is that the patient had a few episodes of AMS and therefore, the fluctuating levels of consciousness could have contributed to the lack of pain perception. However, this might be a less likely scenario as a full physical exam and a thorough review of systems were performed at various times by several different members of the medical team, which showed that the patient truly did not experience any pain subjectively or on palpation. Another theory is that this patient had a debilitating course of uncontrolled diabetes that could lead to neuropathy and present with a loss of pain perception. Certain studies have shown the effect of glucose on reducing levels of pain perception [15-16]. Therefore, more research is needed to identify the etiology underlying the lack of pain perception in patients with the painless presentation of calciphylaxis. 

## Conclusions

This patient’s presentation of painless eschars was atypical. Therefore, a high degree of clinical suspicion in patients with ESRD on HD and T2DM is needed to effectively reach a diagnosis of calciphylaxis. The high rate of mortality and morbidity coincide with the risk factors associated with this condition. Early recognition of calciphylaxis in a patient with no pain despite the prevalence of ulcerations warrants a closer look into their comorbidities to identify this disease on the differential as it is critical to start treatment early to prevent devastating outcomes.
